# The extent of linkage disequilibrium in beef cattle breeds using high-density SNP genotypes

**DOI:** 10.1186/1297-9686-46-22

**Published:** 2014-03-24

**Authors:** Laercio R Porto-Neto, James W Kijas, Antonio Reverter

**Affiliations:** 1 CSIRO Food Futures Flagship, 306 Carmody Road, St Lucia, Brisbane QLD 4067, Australia; 2 CSIRO Animal, Food and Health Science, 306 Carmody Road, St Lucia, Brisbane, QLD 4067, Australia

## Abstract

**Background:**

The extent of linkage disequilibrium (LD) between molecular markers impacts genome-wide association studies and implementation of genomic selection. The availability of high-density single nucleotide polymorphism (SNP) genotyping platforms makes it possible to investigate LD at an unprecedented resolution. In this work, we characterised LD decay in breeds of beef cattle of taurine, indicine and composite origins and explored its variation across autosomes and the X chromosome.

**Findings:**

In each breed, LD decayed rapidly and r^2^ was less than 0.2 for marker pairs separated by 50 kb. The LD decay curves clustered into three groups of similar LD decay that distinguished the three main cattle types. At short distances between markers (< 10 kb), taurine breeds showed higher LD (r^2^ = 0.45) than their indicine (r^2^ = 0.25) and composite (r^2^ = 0.32) counterparts. This higher LD in taurine breeds was attributed to a smaller effective population size and a stronger bottleneck during breed formation. Using all SNPs on only the X chromosome, the three cattle types could still be distinguished. However for taurine breeds, the LD decay on the X chromosome was much faster and the background level much lower than for indicine breeds and composite populations. When using only SNPs that were polymorphic in all breeds, the analysis of the X chromosome mimicked that of the autosomes.

**Conclusions:**

The pattern of LD mirrored some aspects of the history of breed populations and showed a sharp decay with increasing physical distance between markers. We conclude that the availability of the HD chip can be used to detect association signals that remained hidden when using lower density genotyping platforms, since LD dropped below 0.2 at distances of 50 kb.

## Background

Linkage disequilibrium (LD) between molecular markers reflects the correlation between genotypes of two markers or the degree of non-random association between their alleles. Previous studies that used single nucleotide polymorphisms (SNPs) to describe patterns of LD in cattle at the whole-genome level [1-6] have suggested that 30 000 to 300 000 SNPs are necessary to perform a genome-wide association study (GWAS), depending on the trait studied and the statistical power desired [1,2]. Today, the availability of high-density SNP platforms that can assay more than 0.5 million loci offers the required marker density.

The extent of LD has implications for both GWAS and the delivery of accurate genomic predictions. However, its importance is often neglected despite the fact that it is known that it can introduce bias. Collecting and using SNP genotyping data have exploded for cattle in the last few years due in part to decreasing genotyping cost and to efforts to improve cattle breeding through genomic selection. Despite this, few studies have documented the behaviour of LD using the expanded set of 777 000 SNPs available on the BovineHD platform (Illumina Inc, San Diego). One of the significant advances of this denser chip is that it allows for an accurate estimation of LD over short physical distances as it contains many more marker pairs separated by 10 kb or less.

Here, we present the LD decay curves for SNPs on bovine autosomes and the X chromosome for three genetic groups of cattle breeds: *Bos taurus* (taurine), *Bos indicus* (indicine) and a composite beef cattle group. The results were compared to an independent population to confirm and potentially generalize the findings. This report is intended to be used as an updated description of the extent of LD in beef cattle.

## Methods

All analyses were performed using genotypes generated in previous work. Therefore, for this study, no animal ethics approval was requested because no new animals were sampled.

Animals used in this study (Table 1) were part of a large experimental Australian population [7] that includes the three main cattle types: *Bos taurus* breeds (Angus, Hereford, Limousin and Shorthorn), *Bos indicus* (Brahman) and composite cattle (Tropical Composite, Santa Gertrudis and Belmont Red). To confirm our findings, genotyping data from each cattle type (Angus, Brahman and Santa Gertrudis) were sourced from the Bovine HapMap consortium [3].

**Table 1:** Description of samples and summary of results^*^

**Breed**	**Breed type**	**Total nb**	**Nb of males**	***Pn***	***He***	**LD at 10 kb autos**	**LD at 10 kb BTAX**	**LD at 70 kb autos**	**LD at 70 kb BTAX**
***Australian population***									
Angus	Bt	195	165	0.85	0.27	0.46	0.47	0.20	0.25
Hereford	Bt	79	73	0.85	0.31	0.49	0.51	0.23	0.28
Limousin	Bt	62	58	0.86	0.30	0.42	0.49	0.15	0.23
Shorthorn	Bt	130	127	0.90	0.25	0.43	0.46	0.19	0.27
Tropical Composite	Bt × Bi	351	186	1.00	0.35	0.30	0.43	0.13	0.33
Santa Gertrudis	Bt × Bi	168	82	0.99	0.33	0.32	0.47	0.16	0.37
Belmont Red	Bt × Bi	97	77	1.00	0.34	0.33	0.44	0.15	0.34
Brahman	Bi	519	304	1.00	0.26	0.25	0.42	0.13	0.32
***Bovine HapMap population***									
Angus	Bt	55	42	0.84	0.29	0.46	0.47	0.20	0.23
Santa Gertrudis	Bt × Bi	35	32	0.98	0.34	0.34	0.48	0.18	0.39
Brahman	Bi	46	36	0.91	0.25	0.28	0.43	0.16	0.33

^*^Bt = *Bos taurus*; Bi = *Bos indicus*; Bt × Bi = composite breed; *Pn* = proportion of polymorphic SNPs; *He* = gene diversity or heterozygosity; 10 kb = average r^2^ of all pairs of SNPs that are between 9.5 to 10.5 kb apart; Autos = autosomes; 70 kb = average r^2^ of all pairs of SNPs that are between 69.5 to 70.5 kb apart.

All animals were genotyped using the BovineHD SNP chip (Illumina, San Diego; http://www.illumina.com/documents/products/datasheets/datasheet_bovineHD.pdf) that includes 777 962 markers. Quality control and imputation of missing data in the Australian sample followed the pipeline described by Bolormaa et al. [8]. Briefly, stringent filters were applied to each SNP (call rate, duplicated map position, extreme departure from Hardy-Weinberg equilibrium), resulting in 729 068 informative SNPs. Missing genotypes were imputed within each breed type using 30 iterations of the BEAGLE software [9]. Genotypes for the same set of SNPs were extracted from the Bovine HapMap dataset [10] but missing genotypes were not imputed. LD between each pair of SNPs, measured as r^2^, which is less susceptible to bias due to differences in allelic frequency [4], and within-breed genetic diversity (heterozygosity and proportion of polymorphic SNPs) were calculated using PLINK v1.07 [11]. For the X chromosome, two scenarios were explored: one including all markers, and the second including only fairly polymorphic markers with a minor allele frequency (MAF) greater than 0.1 in all breeds.

## Results and discussion

A high proportion of polymorphic markers was observed across all breeds, with the taurine breeds showing a slightly lower proportion (*Pn* ~ 0.86) than their indicine and composite counterparts (*Pn* ~ 1.00 for both) (Table 1). Heterozygozity (*He*) ranged from 0.25 (Brahman from the HapMap dataset and Shorthorn) to 0.35 (Tropical Composite). In general, the composite breeds showed higher *He* (0.34) than the taurine (0.28) and indicine breeds (0.26) because they originated from a mixture of both these types of cattle.

**Figure 1:**
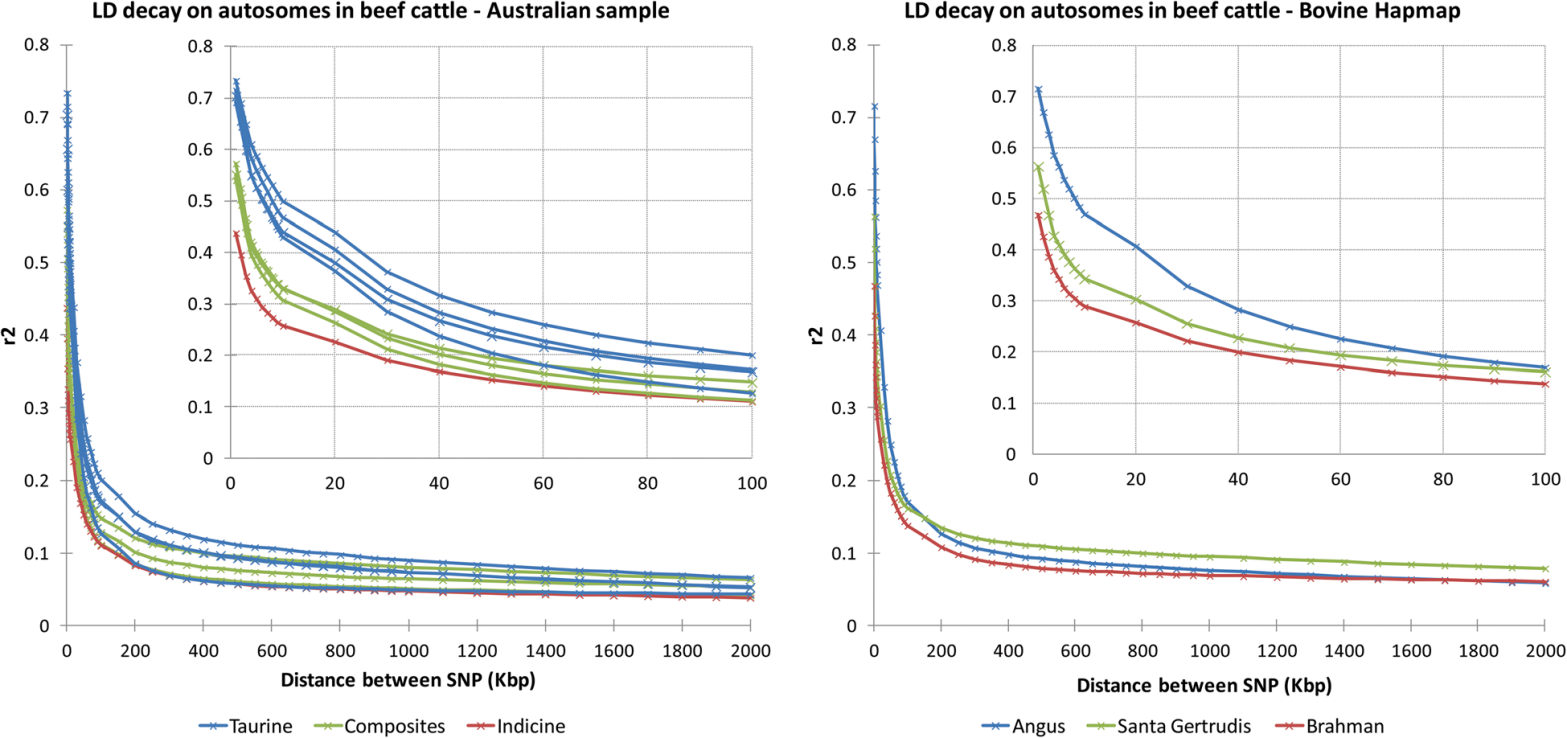
**Linkage disequilibrium (r**^**2**^**) decay on beef cattle autosomes using a selection of Australian cattle breeds and HapMap samples.** Additional file 1: Figure S1 discriminates each breed.

The pattern of LD differed between breeds and the resulting decay curves could be grouped according to breed type (Figure 1 and Additional file 1: Figure S1). At short marker distances, indicine breeds had lower LD for autosomes compared to either the composite (intermediate) or taurine (highest) breeds. This is in agreement with previous studies [2,3], but the degree of variation fluctuates. When the distance between markers was 10 kb, the average observed LD (r^2^) for Brahman and Angus was 0.25 and 0.46, respectively (Table 1), which is equivalent to the LD reported for a comparable indicine cattle breed i.e. Nelore (0.27) [12], but higher than the value previously reported for Angus (0.35) [13]. This difference is not as clear for markers separated by a larger physical distance (> 70 kb) where LD quickly approached background levels, and r^2^ was ~ 0.10 in both studies and also in dairy breeds [6]. The average LD between unlinked markers (SNPs on different chromosomes) was at the background level or less across all breeds (see Additional file 2: Table S1) and was negatively correlated with sample size (Pearson correlation, r = -0.75). Indicine cattle continued to have a lower LD than most of the other breeds when the distances between markers were large, which suggests that they originated from a larger ancestral population.

**Figure 2:**
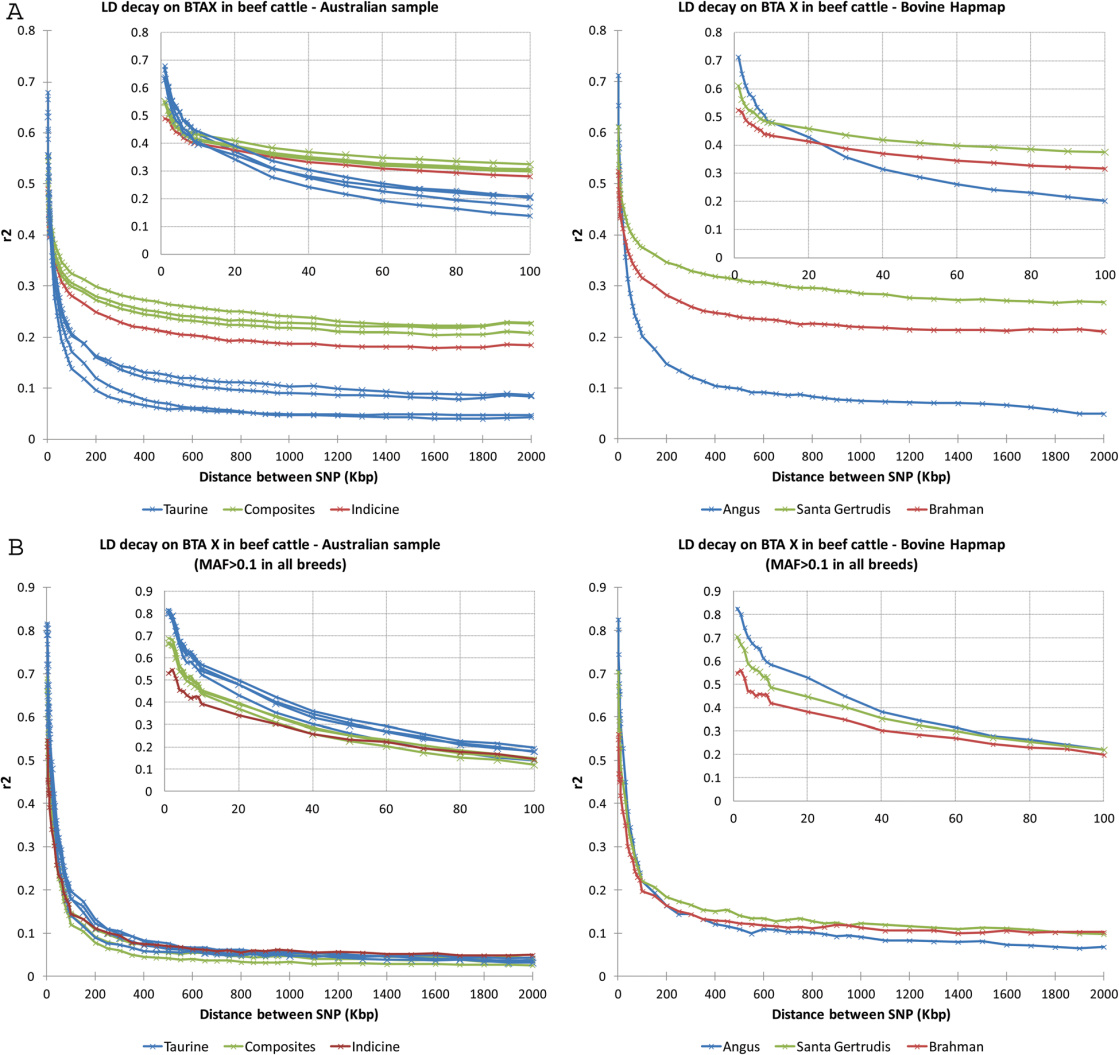
**Linkage disequilibrium (r**^**2**^**) on the X chromosome of beef cattle using all SNPs (A) and only polymorphic SNPs in all breeds (B).** MAF = minor allele frequency.

Analysis of LD across the bovine X chromosome (BTAX) revealed a different pattern to that observed for autosomes (Figure 2). The LD decay curves were still grouped by cattle type, however with a different ranking compared to what was observed for LD on autosomes. Over very short distances between markers on BTAX (< 5 kb), the indicine breeds still had the lowest average LD (r^2^ ~ 0.5) and the taurine breeds had the highest (r^2^ > 0.6). However, contrary to the pattern observed for autosomes, LD across BTAX decayed fastest in the taurine breeds, such that for marker pairs separated by 50 kb, the average LD was lower than that in either of the composite indicine populations (Figure 2A). The same LD patterns were observed when males only were evaluated (see Additional file 1: Figure S2). However, when only SNPs that were polymorphic for all breeds (MAF > 0.1) were used, the LD decay for BTAX became much more homogeneous across all breeds and, in fact, did not differ much from the results obtained for autosomes (Figure 2B). Because of the bottlenecks that cattle populations have experienced since their domestication and more recently during breed formation and because of the frequent intensive use of artificial insemination, it would be reasonable to expect extensive LD on BTAX. This expectation agrees with the LD decay observed for indicine and composite breeds when using all SNPs but not with the LD decay observed for all taurine breeds, nor for the LD decay observed for all breeds when only polymorphic SNPs were used. We speculate that the use of all markers inflated the LD observed for indicine and composite breeds (or biased the LD for taurine breeds downwards). However, the use of only polymorphic SNPs was too stringent and did not allow the analyses to capture the expected difference in LD on BTAX due to its unique inheritance.

To assess whether the results obtained here were a specific feature of the Australian population, we repeated the analyses with an independent sample of Angus, Santa Gertrudis and Brahman animals from the Bovine HapMap dataset [3,10]. Results for all analyses on these populations showed high concordance with LD observed in the Australian populations for both the autosomes and BTAX (Figures 1 and 2).

## Conclusions

Our results expand on previous studies of genome-wide LD in bovine populations. By using larger samples and a much higher density of markers than before and by exploring variation across autosomes and the X chromosome, we obtained an exponential increase in pair-wise LD comparisons, which allowed us to produce robust results. Because LD dropped below 0.2 at marker distances above 50 kb, we conclude that the availability of the HD chip enables detection of association signals that remained hidden when using lower density genotyping platforms.

## Competing interests

The authors declare that they have no competing interests.

## Authors’ contributions

LRPN, AR and JWK planned the experiment. LRPN ran the analyses. LRPN, AR and JWK drafted the manuscript. All authors read and approved the final version.

## Supplementary Material

Additional file 1: Figure S1 Linkage disequilibrium (r^2^) decay on beef cattle autosomes from the Australian sample. AA = Angus, BB = Brahman, TC = Tropical Composite, SG = Santa Gertrudis, BR = Belmont Red, HH = Hereford, LL = Limousin, SS = Shorthorn. Plot of the linkage disequilibrium (r^2^) decay on beef cattle autosomes from the Australian sample colour-coded per breed. **Figure S2.** Linkage disequilibrium (r^2^) decay on the X chromosome of male beef cattle only. AA = Angus, BB = Brahman, TC = Tropical Composite, SG = Santa Gertrudis, BR = Belmont Red, HH = Hereford, LL = Limousin, SS = Shorthorn. Plot of the linkage disequilibrium (r^2^) decay on the X chromosome of male beef cattle colour-coded per breed and cattle type. Click here for file

Additional file 2: Table S1 Average linkage disequilibrium (r^2^) between unlinked markers. About ~7 K SNPs were randomly sampled and linkage disequilibrium was calculated for SNP pairs on different chromosomes. Click here for file
