# Supplementary figures and images for: The extent of linkage disequilibrium in beef cattle breeds using high-density SNP genotypes
Source: Genet Sel Evol. 2014 Mar 24;46(1):22. doi: 10.1186/1297-9686-46-22 (PMC4021229; doi:10.1186/1297-9686-46-22)

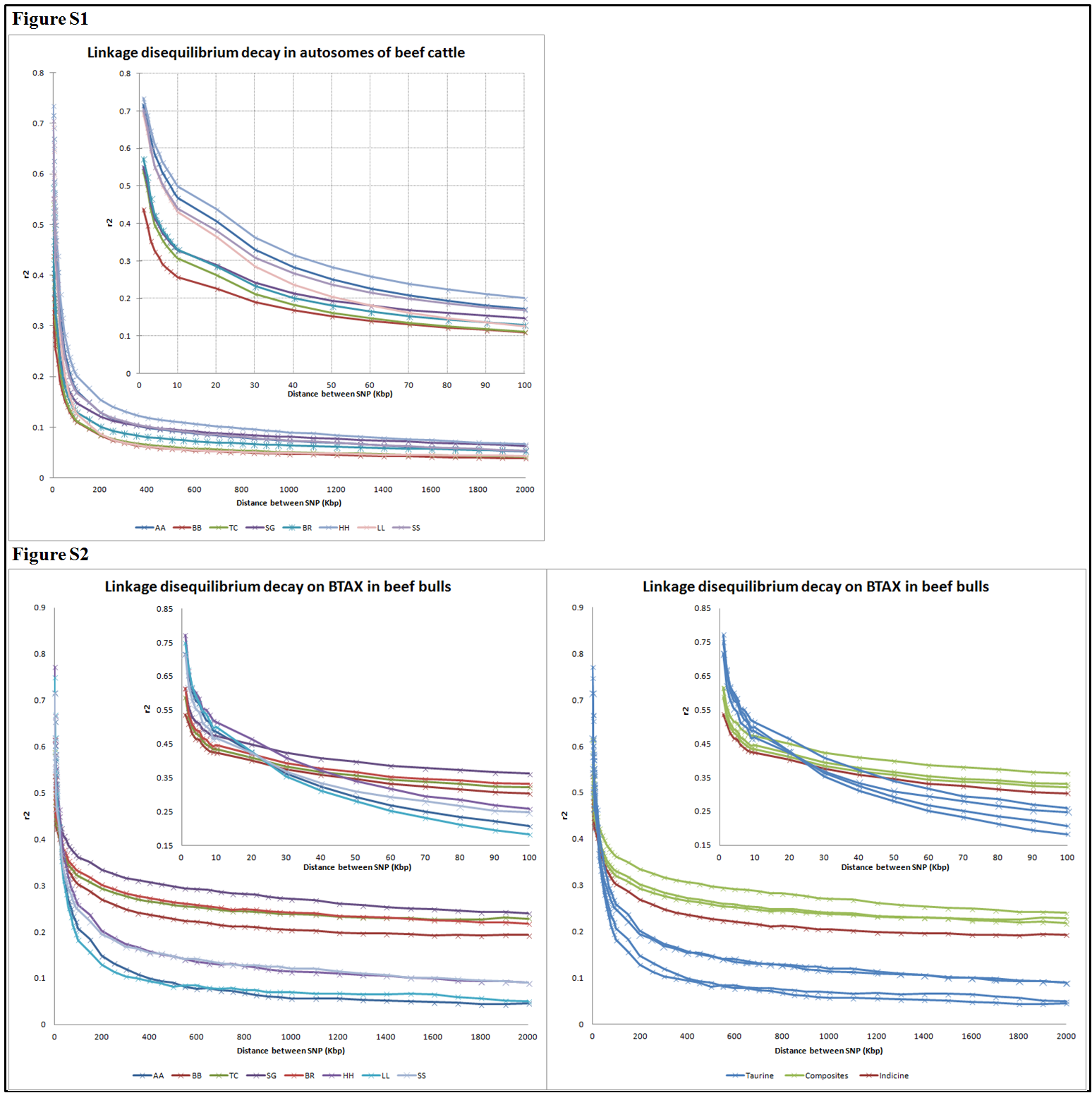

Supplement: Additional file 1: Figure S1 — Linkage disequilibrium (r2) decay on beef cattle autosomes from the Australian sample. AA = Angus, BB = Brahman, TC = Tropical Composite, SG = Santa Gertrudis, BR = Belmont Red, HH = Hereford, LL = Limousin, SS = Shorthorn. Plot of the linkage disequilibrium (r2) decay on beef cattle autosomes from the Australian sample colour-coded per breed. Figure S2. Linkage disequilibrium (r2) decay on the X chromosome of male beef cattle only. AA = Angus, BB = Brahman, TC = Tropical Composite, SG = Santa Gertrudis, BR = Belmont Red, HH = Hereford, LL = Limousin, SS = Shorthorn. Plot of the linkage disequilibrium (r2) decay on the X chromosome of male beef cattle colour-coded per breed and cattle type. [file 1297-9686-46-22-S1.tiff]
