# Supplementary material for: The extent of linkage disequilibrium in beef cattle breeds using high-density SNP genotypes
Source: Genet Sel Evol. 2014 Mar 24;46(1):22. doi: 10.1186/1297-9686-46-22 (PMC4021229; doi:10.1186/1297-9686-46-22)
Supplement: Additional file 2: Table S1 — Average linkage disequilibrium (r2) between unlinked markers. About ~7 K SNPs were randomly sampled and linkage disequilibrium was calculated for SNP pairs on different chromosomes. [file 1297-9686-46-22-S2.docx]

Table S1. Average linkage disequilibrium (r^2^) between unlinked markers – ~7K SNP were randomly sampled and linkage disequilibrium calculated for SNP pairs involving two different chromosomes.

| **Breed** | **Type^*^** | **N of SNP pairs evaluated** | **Average LD unlinked SNP** |
| --- | --- | --- | --- |
| Angus | Bt | 17,961,874 | 0.007 |
| Hereford | Bt | 19,301,446 | 0.019 |
| Limousin | Bt | 18,555,085 | 0.027 |
| Shorthorn | Bt | 20,324,914 | 0.016 |
| Tropical Composite | Bt × Bi | 24,858,688 | 0.005 |
| Santa Gertrudis | Bt × Bi | 24,315,450 | 0.008 |
| Belmont Red | Bt × Bi | 26,297,693 | 0.013 |
| Brahman | Bi | 25,732,196 | 0.003 |

^*^ Bt – *Bos taurus*, Bi – *Bos indicus*, Bt × Bi – composite breed *Bos taurus* x *Bos indicus*.
